# Supplementary material for: Effect of Tibolone on Bone Mineral Density in Postmenopausal Women: Systematic Review and Meta-Analysis
Source: Biology (Basel). 2021 Mar 10;10(3):211. doi: 10.3390/biology10030211 (PMC8000366; doi:10.3390/biology10030211)
Supplement: Supplementary file 1 [file biology-10-00211-s001.pdf]

biology

MDPI

Review

# Effect of Tibolone on Bone Mineral Density in Postmenopausal Women: Systematic Review and Meta-Analysis

**Lizett Castrejón-Delgado [1], Osvaldo D. Castelán-Martínez [2], Patricia Clark [3], Juan Garduño-Espinosa [4], Víctor Manuel Mendoza-Núñez [1] and Martha A. Sánchez-Rodríguez [1,*]**

1 Research Unit on Gerontology, Facultad de Estudios Superiores Zaragoza, Universidad Nacional Autónoma de México, Mexico City 09230, Mexico; lizettcastrejon@hotmail.com (L.C-D.); mendovic@unam.mx (V.M.M.-N.)
2 Clinical Pharmacology Laboratory, Facultad de Estudios Superiores Zaragoza, Universidad Nacional Autónoma de México, Mexico City 09230, Mexico; castelan@unam.mx
3 Clinical Epidemiology Research Unit, Hospital Infantil de México Federico Gómez, Mexico City 06720, Mexico; osteoclark@gmail.com
4 Research Department, Hospital Infantil de México Federico Gómez, Mexico City 06720, Mexico; juan.gardunoe@gmail.com
* Correspondence: masanrod@comunidad.unam.mx; Tel.: +52-55-5623-0700 (ext. 83210)

**Table S1.** Search strategy.

| Database | Search strategy | Items found | Date |
|---|---|---|---|
| MEDLINE | ("tibolone" [Supplementary Concept]) AND ("Bone Density"[Mesh] OR "Osteoporosis, Postmenopausal"[Mesh]) | 135 | July 17,2020 |
| Cochrane library | tibolone AND (bone density OR osteoporosis) | 126 | July 17,2020 |
| ScienceDirect: | Tibolone, bone density | 37 | July 17,2020 |
| Scopus | Tibolone AND "bone density" | 194 | July 17, 2020 |
| Epistemonikos | tibolone AND (bone density OR osteoporosis) | 38 | July 17,2020 |
| Lilacs | Tibolona | 49 | July 17,2020 |
| SciELO | (tibolone) AND (bone) | 4 | July 17,2020 |
| IMBIOMED | Tibolona | 7 | July 17,2020 |
| Medigrafic: | Tibolona | 6 | July 17,2020 |

**Table S2.** Characteristics of the excluded studies (n = 24).

| Reference | Reasons for exclusion |
|---|---|
| Geusens *et al.*, 1991[41] | Patients with fractures |
| Prelevic *et al.*, 1996[42] | Observational study |
| Lippuner K *et al.*, 1996[43] | Duplicated results |
| Park *et al.*, 2001[44] | Full text unreachable |
| Paviainen *et al.*, 1999[45] | Patients with fractures |
| Pavlov *et al.*, 1999[46] | Patients with fractures |
| Rymer *et al.*, 2001[47] | Duplicated results |
| Rymer *et al.*, 2002[48] | Duplicated results |
| Papaioannou, 2002[49] | Commentary |
| Arabi *et al.*, 2003[50] | Duplicated population |
| Roux *et al.*, 2003[51] | Duplicated population |
| Zayas Jaime *et al.*, 2004[52] | Comparator: premenopausal women |
| Boyanov *et al.*, 2005[53] | Do not measure bone mineral density |
| Carvalho Lopes *et al.*, 2005[54] | Full text unreachable |
| Zhou *et al.*, 2005[55] | Full text unreachable |
| Bianco *et al.*, 2006[56] | Cohort study |
| Nie *et al.*, 2006[57] | Full text unreachable |
| Delmas *et al.*, 2008[58] | Raloxifene as comparator |
| Caruso *et al.*, 2011[59] | Observational study |
| Krejovic *et al.*, 2011[60] | Tibolone dose is not indicated |
| Krejovic *et al.*, 2012[61] | Do not measure bone mineral density |
| Gupta *et al.*, 2013[62] | Bilateral oophorectomy |
| Kalder M *et al.*, 2016[63] | Bilateral oophorectomy |
| Sánchez-Rodríguez, 2017[64] | ISRCTN Registry |

**Table S3.** Evidence of adverse events.

| Tibolone vs. Non-Active Controls | | | |
|---|---|---|---|
| **Outcomes** | **Tibolone doses (mg)** | **Studies** | **Risk ratio (95%CI), participants.** |
| Headache | 2.5 | 1[24] | 1.71 (0.19 to 15.40), 55 |
| | 1.25 | 1[24] | 1.67 (0.19 to 14.98), 56 |
| Weight increase | 2.5 | 1[21] | 0.49 (0.04 to 5.31), 302 |
| | 1.25 | 1[21] | 1.45 (0.25 to 8.56), 303 |
| Breast pain | 2.5 | 1[24] | 8.75 (0.53 to 145.58), 55 |
| | 1.25 | 1[24] | 7.38 (0.44 to 124.55), 56 |

| | | | |
|---|---|---|---|
| Vaginal discharge | 2.5 | 1[24] | 1.71 (0.38 to 7.71), 55 |
| | 1.25 | 1[24] | 3.61 (0.90 to 14.42), 56 |
| Edema | 2.5 | 1[24] | 0.12 (0.01 to 2.32), 55 |
| | 1.25 | 1[24] | 0.56 (0.08 to 3.65), 56 |
| Itching of vulvae | 2.5 | 1[24] | Not estimable |
| | 1.25 | 1[24] | 6.24 (0.36 to 107.40), 56 |
| Acne | 2.5 | 1[24] | 0.57 (0.04 to 8.65), 55 |
| | 1.25 | 1[24] | 0.56 (0.04 to 8.41), 56 |
| Moniliasis | 2.5 | 1[21] | 8.76 (1.12 to 68.33), 302 |
| | 1.25 | 1[21] | 3.87 (0.44 to 34.23), 303 |
| Hirsutism | 2.5 | 1[24] | 2.92 (0.15 to 57.90), 55 |
| | 1.25 | 1[24] | 1.70 (0.07 to 39.95), 56 |
| Paraesthesia | 2.5 | 1[24] | 1.75 (0.07 to 41.04), 55 |
| | 1.25 | 1[24] | 1.70 (0.07 to 39.95), 56 |
| Melancholia | 2.5 | 1[24] | 0.19 (0.01 to 4.56), 55 |
| | 1.25 | 1[24] | 0.56 (0.04 to 8.41), 56 |
| Fatigue | 2.5 | 1[24] | 2.92 (0.15 to 57.90), 55 |
| | 1.25 | 1[24] | 2.84 (0.14 to 56.36), 56 |
| Allergy | 2.5 | 1[21] | 0.78 (0.21 to 2.85), 302 |
| | 1.25 | 1[21] | 0.09 (0.00 to 1.58), 303 |
| Hot flashes | 2.5 | 1[21] | 0.29 (0.11 to 0.76), 302 |
| | 1.25 | 1[21] | 0.63 (0.30 to 1.29), 303 |
| Anxiety | 2.5 | 1[21] | 1.30 (0.30 to 5.70), 302 |
| | 1.25 | 1[21] | 4.52 (1.32 to 15.39), 303 |
| Nervousness | 2.5 | 1[21] | 0.42 (0.11 to 1.58), 302 |
| | 1.25 | 1[21] | 0.83 (0.29 to 2.41), 303 |
| Herpes simplex infection | 2.5 | 1[21] | 2.43 (0.48 to 12.35), 302 |
| | 1.25 | 1[21] | 0.19 (0.01 to 4.00), 303 |
| Rhinitis | 2.5 | 1[21] | 0.90 (0.44 to 1.86), 302 |
| | 1.25 | 1[21] | 0.35 (0.13 to 0.94), 303 |
| **Tibolone vs. estrogens** | | | |
| Weight increase | 2.5 | 1[22] | 0.82 (0.49 to 1.37), 149 |
| | 1.25 | 1[22] | 0.89 (0.54 to 1.46), 150 |
| Breast pain | 2.5 | 1[22] | 0.12 (0.03 to 0.48), 149 |
| | 1.25 | 1[22] | 0.11 (0.03 to 0.48), 150 |
| Depression | 2.5 | 1[22] | 0.49 (0.16 to 1.57), 149 |
| | 1.25 | 1[22] | 0.61 (0.21 to 1.78), 150 |
| Acne | 2.5 | 1[22] | 0.65 (0.11 to 3.77), 148 |

## References


1. Geusens, P.; Dequeker, J.; Gielen, J.; Schot, L.P.C. Non-linear increase in vertebral density induced by a synthetic steroid (Org OD 14) in women with established osteoporosis. *Maturitas* **1991**, *13*, 155–162, doi:10.1016/0378-5122(91)90099-C.
2. Prelevic, G.M.; Bartram, C.; Wood, J.; Okolo, S.; Ginsburg, J. Comparative effects on bone mineral density of tibolone, transdermal estrogen and oral estrogen/progestogen therapy in postmenopausal women. *Gynecol. Endocrinol.* **1996**, *10*, 413–420, doi:10.3109/09513599609023606.
3. Lippuner, K.; Hanggi, W.; Birkhäuser, M. Prevention of postmenopausal osteoporosis in a comparative study with tibolone, oral estradiol and transdermal estradiol. *Osteoporos. Int.* **1996**, *6*, 230, doi:10.1007/BF02500456.
4. Park, J.W.; Shin, G.C.; Hur, E.J. The effect of tibolone on bone mineral density in postmenopausal women with osteopenia or osteoporosis. *Korean J. Obstet. Gynecol.* **2001**, *42*, 1324–1327.
5. Parviainen, M.T.; Jääskeläinen, K.; Kröger, H.; Arnala, I.; Alhava, E. Urinary bone resorption markers in monitoring treatment of symptomatic osteoporosis. *Clin. Chim. Acta* **1999**, *279*, 145–154, doi:10.1016/S0009-8981(98)00172-7.
6. Pavlov, P.W.; Ginsburg, J.; Kicovic, P.M.; Van der Schaaf, D.B.; Prelevic, G.; Coelingh Bennink, H.J.T. Double-blind, placebo-controlled study of the effects of tibolone on bone mineral density in postmenopausal osteoporotic women with and without

previous fractures. *Gynecol. Endocrinol.* **1999**, *13*, 230–237, doi:10.3109/09513599909167560.
7. Rymer, J.; Robinson, J.; Fogelman, I. Effects of 8 years of treatment with tibolone 2.5 mg daily on postmenopausal bone loss. *Osteoporos. Int.* **2001**, *12*, 478–483, doi:10.1007/s001980170093.
8. Rymer, J.; Robinson, J.; Fogelman, I. Ten years of treatment with tibolone 2.5 mg daily: Effects on bone loss in postmenopausal women. *Climacteric* **2002**, *5*, 390–398, doi:10.1080/cmt.5.4.390.398.
9. Papaioannou, A. Tibolone at daily doses of 0.625–2.5mg increased bone mineral density in early postmenopausal women. *Evid.-Based Obstet. Gynecol.* **2002**, *4*, 100–101, doi:10.1054/ebog.2002.0044.
10. Arabi, A.; Garnero, P.; Porcher, R.; Pelissier, C.; Benhamou, C.L.; Roux, C. Changes in body composition during post-menopausal hormone therapy: A 2 year prospective study. *Hum. Reprod.* **2003**, *18*, 1747–1752, doi:10.1093/humrep/deg331.
11. Roux, C.; Arabi, A.; Porcher, R.; Garnero, P. Serum leptin as a determinant of bone resorption in healthy postmenopausal women. *Bone* **2003**, *33*, 847–852, doi:10.1016/j.bone.2003.07.008.
12. Zayas Jaime, F.J.; Alanís, L.J.E.; Cueto, M.A.G.; Meras, E.A. Efectos del tratamiento de reemplazo hormonal sobre el sistema cardiovascular, el hueso y la memoria en pacientes en etapa del climaterio. *Ginecol. Obstet. Mex.* **2004**, *72*, 16–22.
13. Boyanov, M.A.; Shinkov, A.D. Effects of tibolone on body composition in postmenopausal women: A 1-year follow up study. *Maturitas* **2005**, *51*, 363–369, doi:10.1016/j.maturitas.2004.09.003.
14. Carvalho-Lopes, C.M. Evaluation of metabolic and anatomic parameters of menopausal women treated with tibolone. *Rev. Bras. Med.* **2005**, *62*, 397–400.
15. Zhou, Y.; Zhou, S. Reaction to hormone replacement therapy in women at perimenopausal period and within and more than 5 years post-menopause: Changes of bone mineral density at lumbar spine and hip. *Chin. J. Clin. Rehabil.* **2005**, *9*, 168–169.
16. Bianco, V.; Murina, F.; Robertti, P.; Valente, I. Tibolone in the treatment of menopause: Compliance, efficacy and safety in a ten year experience. *Minerva Ginecol*. **2006**, *58*, 335–344.
17. Nie, M.; Sun, M.L.; Ge, Q.S. [Effects of long-term and low-dose hormone replacement therapy on bone mineral density in post-menopausal women]. *Zhongguo Yi Xue Ke Xue Yuan Xue Bao* **2006**, *28*, 421–424. (In Chinese)
18. Delmas, P.D.; Davis, S.R.; Hensen, J.; Adami, S.; van Os, S.; Nijland, E.A. Effects of tibolone and raloxifene on bone mineral density in osteopenic postmenopausal women. *Osteoporos Int.* **2008**, *19*, 1153–1160, doi:10.1007/s00198-007-0545-3. .
19. Caruso, S.; Falduzzi, C.; Ventura, B.; Rubino, C.; Ferrara, L.; Polizzi, S.; Grasso, S.; Di Leo, S.; Cianci, A. Ruolo del tibolone nella prevenzione e nella terapia dell'osteoporosi postmenopausale. *Ital. Ostet. Ginecol.* **2011**, *33*, 335–337.
20. Krejovic, S.M.; Zivanovic, A.; Hasani, B.; Ille, T.; Glisic, A. Effects of tibolone on bone mass in postmenopausal women. *Turkish J. Rheumatol.* **2011**, *26*, 226–232, doi:10.5152/tjr.2011.035.
21. Krejović, S.M.; Živanović, A.; Živanović, S.; Marković, R. Effects of tibolone on markers of bone metabolic activity in postmenopausal women. *J. Med. Biochem.* **2012**, *31*, 121–125, doi:10.2478/v10011-011-0050-2.
22. Gupta, B.; Mittal, P.; Khuteta, R.; Bhargava, A. A comparative study of CEE, tibolone, and DHEA as hormone replacement therapy for surgical menopause. *J. Obstet. Gynecol. India* **2013**, *63*, 194–198, doi:10.1007/s13224-012-0297-7.
23. Kalder, M.; Kyvernitakis, I.; Hars, O.; Kauka, A.; Hadji, P. Comparison of combined low-dose hormone therapy vs. tibolone in the prevention of bone loss. *Climacteric* **2016**, *19*, 471–477, doi:10.1080/13697137.2016.1198313.
24. Sánchez-Rodríguez, M.A. Registry ISRCTN14939779. Double-blind placebo controlled trial of tibolone and oestrogen hormone therapy on oxidative stress associated to postmenopausal disturbances in women with early postmenopause: MOS study. *Regist. ISRCTN* **2016**, 14939779, doi:10.1186/ISRCTN14939779.
